# Correlation between Muscle Strength and Muscle Mass, and Their Association with Walking Speed, in Community-Dwelling Elderly Japanese Individuals

**DOI:** 10.1371/journal.pone.0111810

**Published:** 2014-11-03

**Authors:** Itsushi Hayashida, Yoshimi Tanimoto, Yuka Takahashi, Toshiyuki Kusabiraki, Junko Tamaki

**Affiliations:** Department of Hygiene & Public Health, Osaka Medical College, Osaka, Japan; University of Rome Foro Italico, Italy

## Abstract

**Objectives:**

We aimed to assess the correlation between muscle strength and muscle mass based on sex and age, and their association with walking speed, which is a health predictor for independent living, in elderly Japanese individuals.

**Methods:**

The participants included 318 (111 men, 207 women) community-dwelling elderly Japanese individuals aged ≥65 years. Knee extension strength was assessed as an indicator of muscle strength, and bioelectrical impedance analysis was used to measure muscle mass. The maximum walking speed of participants was recorded. All measurements were categorized based on sex and age groups as follows: young-old (age, 65–74 years) and old-old (age, ≥75 years).

**Results:**

Appendicular muscle mass and knee extension strength decreased with age in both men and women. In men, knee extension strength showed significant positive correlations with leg and appendicular muscle mass in both young-old and old-old age groups. However, in women, only the old-old age group showed significant positive correlations between knee extension strength and leg and appendicular muscle mass. Muscle strength was significantly positively correlated with maximum walking speed in all groups, whereas muscle mass was not significantly correlated with maximum walking speed in men and women.

**Conclusions:**

Muscle strength was significantly correlated with muscle mass in both age groups in men. However, in women, the correlation between muscle strength and muscle mass differed according to age. This finding suggests that the relationship between muscle strength and muscle mass differs according to sex and age. Muscle strength showed significant correlation with walking speed in both men and women in both age groups. These findings suggest that it is necessary to recognize that muscle strength is different from muscle mass, and that an individualized approach to prevent decline of muscle strength and muscle mass is necessary for health promotion in elderly.

## Introduction

Japan has the highest proportion of elderly individuals in the world (23.3%, as reported in 2011) with high life expectancy (79.64 years for men and 86.39 years for women, as reported in 2010). This proportion appears to be increasing, as indicated by the increase in the number of subjects using the long-term care insurance system from 2.2 million in 2000 to 4.7 million in 2010 [1]. It is therefore important for the elderly to maintain their independence.

In recent years, on one hand, age-related loss of muscle strength [2] has been widely considered to be a major cause of disability in the elderly [3], and has emerged as one of the most prevalent problems in this population [4], [5]. On the other hand, reduction in muscle mass is also one of the most important age-related changes in the body. Growing evidence shows that reduction in muscle mass contributes to functional disability in elderly individuals [6]–[8]. Studies have examined the relationship between muscle strength and muscle mass [9]–[11] and have found that loss of muscle strength is primarily a direct result of age-associated decline in muscle mass [12]. However, recent evidence questions this relationship, as shown by a study in which muscle mass maintenance or gain did not prevent age-related decrease in muscle strength [9]. To our knowledge, no detailed report has been published thus far on the relationship between muscle strength and muscle mass in Japanese individuals. Muscle strength and muscle mass are affected by ethnicity and lifestyle characteristics [11], [13]. It is necessary to clarify the relationships between muscle strength and muscle mass in Japanese individuals for maintaining muscle condition and promoting health in elderly Japanese individuals.

Walking speed is an objective measurement of lower extremity functions and is a good predictor of functional capacity decline in community-dwelling elderly [14]–[16]. To maintain the independence of elderly individuals, it is useful to determine the association of walking speed with muscular strength and muscle mass.

This study assessed the correlation between muscle strength and muscle mass based on age and sex, and their association with walking speed, which is a health predictor for independent living, in elderly Japanese individuals.

## Subjects and Methods

### Ethics statement

The study protocol was approved by the Osaka Medical College ethics committee, and all participants provided written informed consent.

### Study area and subjects

Takatsuki City, a metropolitan suburb in the north of Osaka Prefecture, is home to 80,695 individuals aged ≥65 years; 22.5% of this population is elderly. In this region, welfare centers for the aged and community centers are the main organizations that provide social support to community-dwelling elderly individuals. A total of 318 people aged ≥65 years, who were registered at or used the centers participating in this study, were included. All measurements were obtained at the community center between May and June 2011.

### Muscle strength measurements

Bilateral isometric knee extension strength was assessed as an indicator of overall muscle strength using a hand-held dynamometer (μTas-01, Anima Co., Tokyo, Japan). The belt has a buckle to fix the sensors, enabling its length to be adjusted, and a plate for attaching the sensors.

Subjects sat upright in a chair with the hips and knees flexed to approximately 90 degrees. The subjects kept their trunks vertical, and their hands were positioned across their chests. The metal sensor area on the hand-held dynamometer has rubber pads covering its surface. Velcro straps attached to the sensor area were used to fix the sensors on the distal anterior surface of the lower thigh, which was the part being measured. The lower edge of the sensor was located at the height of the upper edge of the malleolus medialis. The leg being measured was fixed to the chair leg located behind the subjects' leg. When the subjects' leg was fixed to a chair leg, the flexural angle of the subjects' knee was adjusted to 90 degrees with an adjustable belt attached to the hand-held dynamometer [17]. Isometric movement was conducted by exerting maximum effort for knee joint extension movement for approximately five seconds. Care was taken to ensure that the participants' buttocks did not lose contact with the chair during the measurement. The highest of the 4 values (2 from each side) of knee extension strength was used for each subject.

### Muscle mass measurements

Muscle mass was measured by bioimpedance analysis (BIA) using the Body Composition Analyzer MC-190 (Tanita Corp., Tokyo, Japan) as described previously [18]. This system applies electricity at frequencies of 5, 50, 250, and 500 kHz through the body. Whole-body impedance was measured using the ipsilateral foot-hand electrical pathway. BIA has previously been shown to be a safe, convenient, reliable, and valid technique for measuring body composition [19]–[21]. In addition, the equipment is portable, and the test is inexpensive. Further, the results of the present study were consistent with those of previous studies using different measurement techniques, indicating the high reproducibility of BIA results [22], [23]. The recommended BIA measurement conditions were explained to the subjects: (1) fasting for 4 h and no alcohol intake for 8 h before measurement; (2) voiding of the bladder before measurement; and (3) avoiding exercise for 8 h before measurement [19], [24]. Appendicular muscle mass was calculated as the sum of the muscle mass of the arms and legs.

### Maximum walking speed assessment

To assess maximum walking speed, participants were asked to walk straight ahead for 11 m at their maximum speeds. Walking speed was derived from the middle 5 m segment [25]. This test was repeated twice, and the higher speed was considered.

### Statistical analysis

Since the measured values differed between men and women, the measurements were categorized into 2 groups according to sex. The measurements were further categorized according to age into 2 groups: 65–74 years old (young-old subjects) and ≥75 years old (old-old subjects) for each sex. Student's *t*-test was used to analyze the differences between sexes and between age groups. The partial correlation coefficient adjusted by age was used to analyze the relationship between knee extension strength and leg and appendicular muscle mass, and the partial correlation coefficient adjusted by age, height and weight was used to examine the correlation between maximum walking speed, and knee extension strength and muscle mass. All analyses were performed using the SPSS 19.0 package (SPSS, Chicago, IL). A p-value of <0.05 was considered statistically significant.

## Results

**Figure 1:**
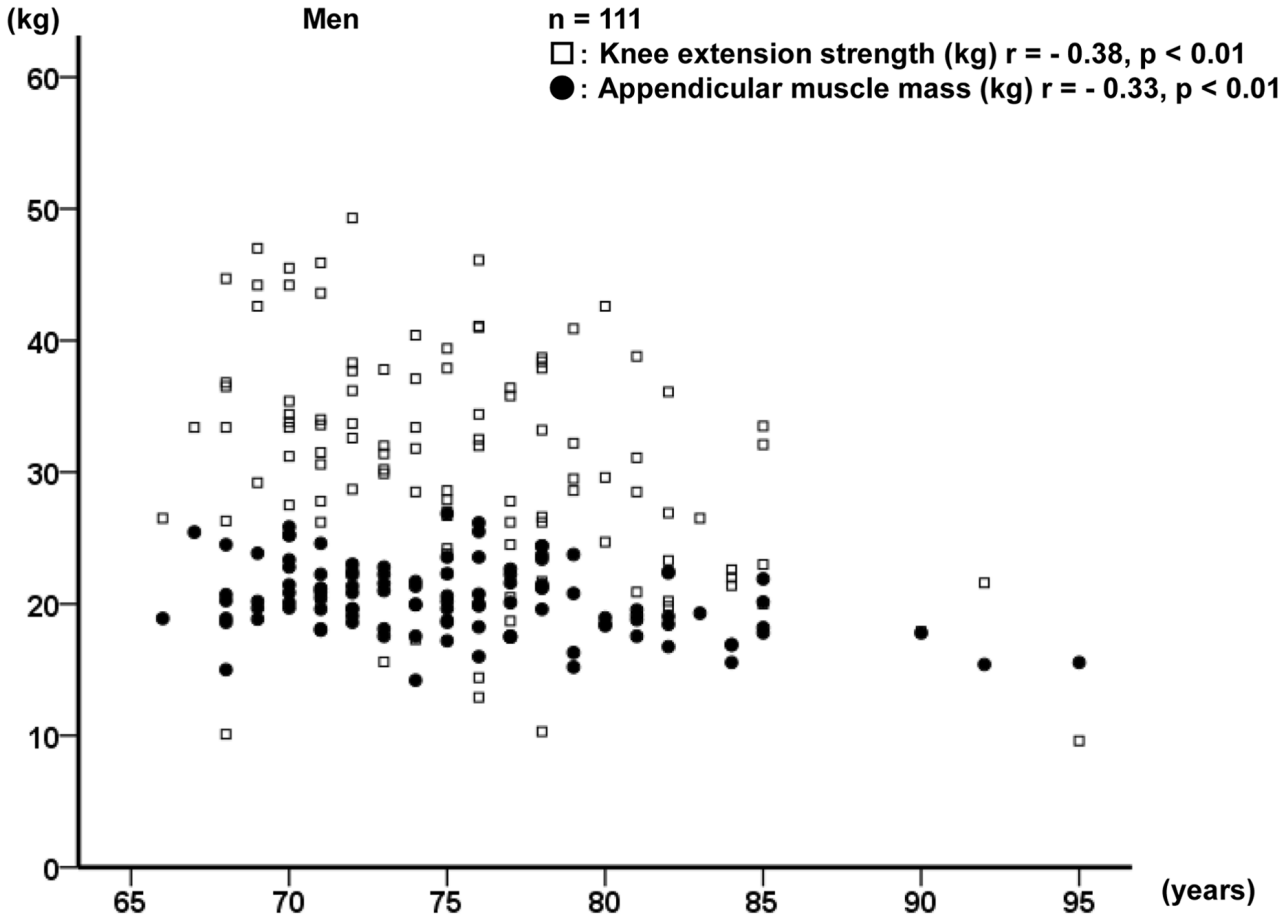
Relationships of age with knee extension strength and appendicular muscle mass in men.

**Figure 2:**
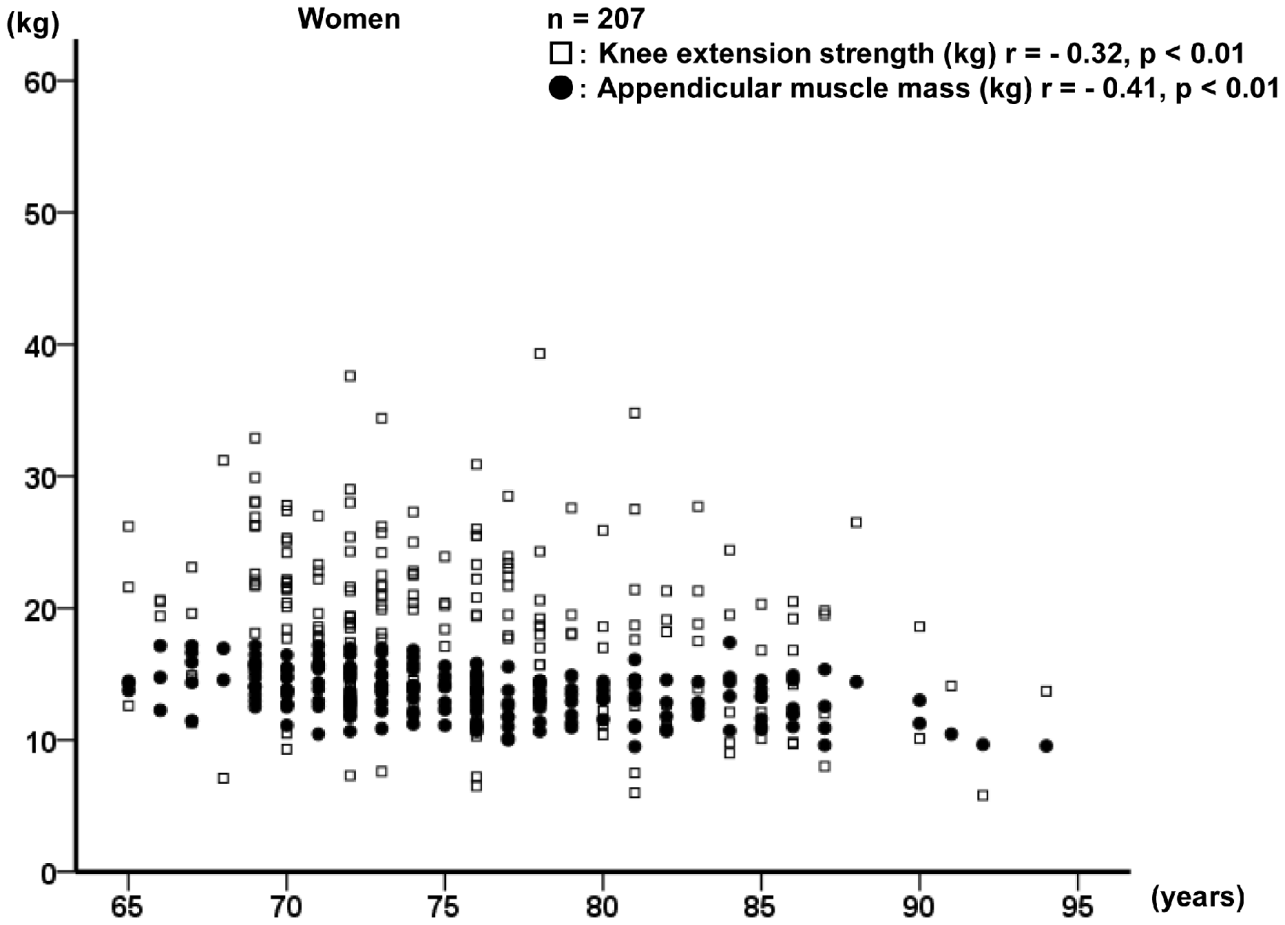
Relationships of age with knee extension strength and appendicular muscle mass in women.

Relationships of age with appendicular muscle mass and knee extension strength for men and women are shown in Figs. 1 and 2, respectively. Overall, the appendicular muscle mass and knee extension strength were found to decrease with age in both men and women.

Table 1 shows the subject characteristics according to sex and age. For all anthropometric measurements, men had significantly higher values as compared to women. The young-old group had higher values of knee extension strength and maximum walking speed than the old-old group in both men and women. Furthermore, muscle mass showed significantly higher values in the young-old group than in the old-old group only among women.

**Table 1:** Characteristics of the study subjects according to sex and age.

	Men			Women		
Variable	Total (n = 111)	65–74 years (n = 51)	≥75 years (n = 60)	Total (n = 207)	65–74 years (n = 102)	≥75 years (n = 105)
**Years**	75.5±5.5	70.9±2.0	79.5±4.3	75.8±6.0	70.9±2.3	80.6±4.4
**Height (cm)**	162.3±6.5^b^	162.5±6.0	162.2±6.9	149.1±5.5	151.0±4.7***	147.2±5.6
**Weight (kg)**	61.8±7.6^b^	62.4±7.6	61.3±7.6	50.4±7.6	52.4±7.3***	48.6±7.5
**Body mass index (kg/m^2^)**	23.5±2.8^a^	23.7±3.0	23.3±2.7	22.7±2.9	22.9±2.7	22.4±3.1
**Knee extension strength (kg)**	30.4±8.6^b^	33.0±8.2**	28.1±8.3	19.0±6.2	20.5±5.7***	17.4±6.2
**Leg muscle mass (kg)**	15.7±2.1^b^	16.0±2.0	15.3±2.2	10.5±1.4	11.1±1.3***	10.0±1.3
**Appendicular muscle mass (kg)**	20.4±2.7^b^	20.8±2.5	20.0±2.8	13.5±1.8	14.2±1.6***	12.8±1.7
**Maximum walking speed (m/s)**	1.8±0.3^b^	1.9±0.3*	1.7±0.4	1.6±0.3	1.7±0.2***	1.4±0.3

Values are presented as mean ± standard deviation (SD).

*: p<0.05,

**: p<0.01,

***: p<0.001 (65–74 years vs. ≥75 years).

a : p<0.05,

b : <0.001 (total men vs. women).

The correlation coefficients of muscle strength and muscle mass in men and women are given in Table 2. In men, knee extension strength showed significant positive correlations with leg and appendicular muscle mass in both young-old and old-old age groups. However, in women, only the old-old age group showed significant positive correlations between knee extension strength and leg and appendicular muscle mass.

**Table 2:** Partial correlation coefficients between muscle strength and muscle mass.

	Knee extension strength					
	Men			Women		
	Total (n = 104)	65–74 years (n = 51)	75–84 years (n = 53)	Total (n = 184)	65–74 years (n = 102)	75–84 years (n = 82)
**Leg muscle mass (kg)**	0.33**	0.41**	0.28*	0.22**	0.16	0.29**
**Appendicular muscle mass (kg)**	0.34**	0.41**	0.29*	0.23**	0.16	0.31**

Adjusted by age.

*: p<0.05,

**: p<0.01.

The partial correlation coefficient of maximum walking speed and knee extension strength or muscle mass in men and women are given in Table 3. Maximum walking speed showed a significant positive correlation with knee extension strength in men and women in both age groups. However, leg and appendicular muscle mass had no significant correlation with maximum walking speed in both men and women.

**Table 3:** Partial correlation coefficients between maximum walking speed, and muscle strength and muscle mass.

	Maximum walking speed					
	Men			Women		
	Total (n = 111)	65–74 years (n = 51)	≥75 years (n = 60)	Total (n = 207)	65–74 years (n = 102)	≥75 years (n = 105)
**Knee extension strength (kg)**	0.38**	0.29*	0.42**	0.45**	0.47**	0.42**
**Leg muscle mass (kg)**	0.09	−0.12	0.14	0.05	0.02	0.11
**Appendicular muscle mass (kg)**	0.11	−0.13	0.18	0.07	0.04	0.19

Adjusted by age and height and weight.

*: p<0.05,

**: p<0.01.

## Discussion

The present study showed that muscle strength and muscle mass decrease with age, and have a significant positive correlation with each other in community-dwelling elderly Japanese men and women. Further analysis of muscle strength and muscle mass by age showed a significant correlation only in the old-old age group in women, whereas both young-old and old-old age groups showed a significant correlation in men. These findings suggest that the relationship between muscle strength and muscle mass differs according to sex and age.

The decline in muscle strength and muscle mass is a serious problem in the elderly. The former is known as “dynapenia,” [26] and this condition is closely associated with subsequent physical disability [27], [28]. The Leiden 85-plus study showed that decreased strength is a predictor of an accelerated decline in the ability to perform activities of daily living (ADLs), and in cognition [29]. The Health, Aging, and Body Composition study found that the incidence of disability was higher in the lowest than in the highest quartile of quadriceps strength [26].

Loss of muscle mass that occurs with advancing age, known as sarcopenia, is a serious health issue in the elderly, as it may lead to functional disability, such as dependence in performing ADLs or instrumental ADLs (IADLs) [12], [30]; growing evidence shows that sarcopenia contributes to functional disability in elderly individuals [6], [31]. We have also shown in a previous study that sarcopenia was correlated with IADL disability in elderly Japanese men [32].

Thus, preventing the occurrence of dynapenia or sarcopenia is very important for maintaining functional ability, and both conditions should be considered as significant risk factors for a decline in the ability to perform ADLs and IADLs. Therefore, it is important to reveal the relationship between muscle strength and muscle mass.

A previous study of 200 Americans aged 45–78 years showed that although both muscle strength and muscle mass decreased with aging, the decrease in muscle strength was mainly attributed to the reduction in muscle mass [10]. However, a 5-year longitudinal study of 1678 elderly Americans (Caucasian and those of African descent) aged 70–79 years showed that the age-related loss of muscle strength was greater than that of muscle mass and that the reduction in muscle strength and muscle mass did not occur in parallel [9]. The present study found a significant correlation between muscle mass and muscle strength in Japanese men and women aged ≥65 years. However, when examined according to age, muscle strength showed no relationship with muscle mass in young-old women. It is known that muscle strength is attributed to a combination of “neural” and “muscular” factors and that women cannot increase muscle mass over a limited volume—that is, to increase muscle strength in women, increased neural activity is required to recruit the required number of muscle fibers, and this neural activity decreases with aging [33]. This might explain the lack of a relationship between knee extension strength and muscle mass in young-old women. Further, since neuronal activity declines with age [34], muscle mass may have a greater influence on muscle strength with increasing age, and this may explain the significant relationship between muscle strength and muscle mass noted in the old-old women in the present study. However, in the present study, the partial correlation coefficient was between 0.25 and 0.41; it may be that the other factor affecting muscle strength is neural in origin. Additional studies are thus needed to determine the relationship between muscle strength and muscle mass in a larger sample.

Previous studies have examined the physical predictors of functional capacity decline among the elderly. Among these predictors, walking speed was found to be a good predictor of functional capacity decline in community-dwelling elderly [14]–[16] and is used to screen for functional capacity decline. Some studies reported that muscle strength (leg extensor power) has a significant positive correlation with walking speed in elderly men and women [35]–[37]. Our study results regarding the relationship between muscle strength and walking speed in different age groups were in agreement with those of previous studies. However, muscle mass was not correlated with walking speed. Our previous study showed that there was no significant correlation between muscle mass and walking speed in 395 community-dwelling elderly individuals [38]. Good balance is required for maintaining walking posture [39]. This may be the reason why the correlation between muscle strength and muscle mass was not reflected in their relationship with walking speed. A longitudinal study that shows the validity of this relationship between muscle strength and muscle mass, and walking speed is needed.

Our study has certain limitations. The first is its cross-sectional design. Thus, all findings obtained in this study should be confirmed by a future prospective study. Second, although BMI values (23.5±2.8 kg/m^2^ and 22.7±2.9 kg/m^2^, in men and women, respectively) of our subjects were similar to the average BMI in Japanese individuals (23.2±2.96 kg/m^2^ and 22.9±3.54 kg/m^2^, in men and women, respectively [40]), we believe that they were in relatively good health at approximately 75 years, considering that they could walk by themselves to the study center. Thus, our study population may not accurately represent the general elderly Japanese population.

In summary, our study showed that the relationship between muscle strength and muscle mass in the elderly Japanese population differed according to sex and age. That is, only young-old women showed no significant relation between muscle strength and muscle mass. In addition, muscle strength was related to walking speed in both men and women. However, muscle mass showed no correlation with walking speed in both men and women. Muscle strength was significantly correlated with muscle mass in both age groups in men. However, in women, the correlation between muscle strength and muscle mass differed according to age. This finding suggests that the relationship between muscle strength and muscle mass differs according to sex and age. Muscle strength showed a significant correlation with walking speed in both men and women in both age groups. These findings suggest that it is necessary to recognize that muscle strength is different from muscle mass, and that an individualized approach to prevent decline of muscle strength and muscle mass is necessary for health promotion in elderly.
